# Opioids exacerbate inflammation in people with well-controlled HIV

**DOI:** 10.3389/fimmu.2023.1277491

**Published:** 2023-11-01

**Authors:** Christine M. Dang, C. Mindy Nelson, Daniel J. Feaster, Alexander Kizhner, David W. Forrest, Nobuyo Nakamura, Akshay Iyer, Priya P. Ghanta, Dushyantha T. Jayaweera, Allan E. Rodriguez, Rajendra N. Pahwa, Hansel E. Tookes, Suresh Pallikkuth, Savita G. Pahwa

**Affiliations:** ^1^Department of Microbiology and Immunology, University of Miami, Miller School of Medicine, Miami, FL, United States; ^2^Department of Public Health Sciences, University of Miami, Miller School of Medicine, Miami, FL, United States; ^3^Department of Medicine, Division of Infectious Diseases, University of Miami, Miller School of Medicine, Miami, FL, United States

**Keywords:** opioid use disorder, HIV, inflammation, immune activation, immune senescence

## Abstract

**Introduction:**

People with HIV (PWH) are known to have underlying inflammation and immune activation despite virologic control. Substance use including opioid dependence is common in this population and is associated with increased morbidity and reduced lifespan. The primary objective of the present study termed opioid immunity study (OPIS), was to investigate the impact of chronic opioids in PWH.

**Methods:**

The study recruited people with and without HIV who had opioid use disorder (OUD). Study participants (n=221) were categorized into four groups: HIV+OP+, n=34; HIV-OP+, n=66; HIV+OP-, n=55 and HIV-OP-, n=62 as controls. PWH were virally suppressed on ART and those with OUD were followed in a syringe exchange program with confirmation of OP use by urine drug screening. A composite cytokine score was developed for 20 plasma cytokines that are linked to inflammation. Cellular markers of immune activation (IA), exhaustion, and senescence were determined in CD4 and CD8 T cells. Regression models were constructed to examine the relationships of HIV status and opioid use, controlling for other confounding factors.

**Results:**

HIV+OP+ participants exhibited highest inflammatory cytokines and cellular IA, followed by HIV-OP+ for inflammation and HIV+OP- for IA. Inflammation was found to be driven more by opioid use than HIV positivity while IA was driven more by HIV than opioid use. In people with OUD, expression of CD38 on CD28-CD57+ senescent-like T cells was elevated and correlated positively with inflammation.

**Discussion:**

Given the association of inflammation with a multitude of adverse health outcomes, our findings merit further investigations to understand the mechanistic pathways involved.

## 1 Introduction

Opioid misuse is a worldwide problem with approximately 3 million people in the US and 16 million people globally living with opioid use disorder (OUD). One third of people with OUD administer drugs via injection, a risk factor for infectious complications (1, 2) and risk for HIV transmission by sharing needles or unprotected sex (3–5). About one in ten people with HIV (PWH), suffer from OUD (2). It is well documented that despite virologic control with antiretroviral therapy (ART), PWH can manifest increased cellular immune activation (IA) and excessive inflammation (6) that are considered to be a leading cause of accelerated aging, co-morbidities, and functional immune abnormalities (7, 8). Substance use and addictive disorders are also associated with systemic inflammation that could impact the onset and progression of various diseases (9, 10). In animal models, chronic opioid use increases inflammation and immune activation (11). Whether chronic opioid use exacerbates systemic IA and inflammation in PWH is not known. To delineate if chronic OP use impacts the immune system in PWH we conducted a study termed OPIS (OPioid Immunity Study) for which we enrolled PWH with OUD. To understand the independent effects of OUD and HIV, we included people without HIV (PWoH) with OUD, as well as PWH without OUD, and PWoH without OUD. Independent and interactive effects of opioids and HIV as well as immune senescence and immune exhaustion were examined. This understanding is important for developing therapeutic strategies because of the known deleterious effects of inflammation and immune activation on health outcomes.

PWH and PWoH with OUD were recruited from the syringe services program (SSP) of the Infectious Diseases Elimination Act (IDEA) clinic in Miami that provides general health and HIV care including ART to people with history of OUD as well as syringe exchange and follow up for people undergoing medication-assisted treatment (12). Comparison groups without OUD consisting of PWH and PWoH were recruited from the Infectious Diseases clinics and community outreach. Opioids were the focus of the study, but we took into consideration concomitant use of other drugs including stimulants and applied rigorous statistical methods to control for confounding factors. The study revealed that polydrug use was frequent in this population. The overlapping and distinctive characteristics of HIV and OUD as well as impact of other drugs on the immune system in the study population were ascertained.

## 2 Materials and methods

### 2.1 Study participants

Study participants (n = 221, **Table 1**) were recruited (**Supplementary Figure 1A**) based on HIV-1 status (HIV+/-) and OUD status (OP+/-) into four study groups: HIV+OP+; HIV-OP+; HIV+OP-; and HIV-OP-. Participants ranged from 24-68 years of age. The inclusion criteria required PWH to have been on ART for 6 months or more with plasma HIV RNA <200 copies/mL. OP+ participants were required to have been using opioids for 90 days or more and to test positive for opioids on urine drug testing at study visits. Urine samples were collected in 14-drug urine drug screen (UDS) cups to ascertain concomitant use of other drugs (listed in **Table 2**, 12panelnow.com). Clinical data, nature of opioid drugs, other drugs and responses to questionnaires were collected in a REDCap Database (13). Since recruitment of HIV+OP+ was most arduous, we used this group to drive demographic decisions for recruitment of matching people in other groups as closely as possible. The study period was from September 2020 - May 2022. We used rigorous statistical models throughout the study to control the confounding effect of the demographic differences between the study groups on study outcomes. Peripheral venous blood was collected in heparin tubes, processed for plasma and peripheral blood mononuclear cells (PBMCs) as previously described (14), and cryopreserved until used (**Supplementary Figure 1B**). Additional samples were collected subsequently for other study objectives which are not included in this manuscript.

**Table 1:** Demographic characteristics of the study groups.

Population	HIV+OP+	HIV-OP+	HIV+OP-	HIV-OP-	Comparison among groups
*N* = 221	34	65	59	63	
Median Age in Years (Range)	46 (28-63)	39 (24-63)	52 (32-68)	47 (24-60)	p < 0.001^a^
Gender – n (%)					p < 0.05^b,†^
Female	13 (38%)	15 (24%)	27 (46%)	30 (47%)	
Male	21 (62%)	49 (75%)	29 (49%)	34 (53%)	
Transgender Woman	0 (0%)	0 (0%)	3 (5%)	0 (0%)	
Race – n (%)					p < 0.0001^b,†^
White or Caucasian	25 (74%)	50 (77%)	21 (36%)	19 (30%)	
Black or African-American	5 (15%)	12 (18%)	36 (61%)	40 (63%)	
Native American	1 (3%)	0 (0%)	0 (0%)	0 (0%)	
Native Hawaiian	1 (3%)	1 (2%)	0 (0%)	0 (0%)	
Other Race	2 (6%)	2 (3%)	2 (3%)	4 (6%)	
Ethnicity – n (%)					NS^b^
Hispanic or Latino	14 (41%)	18 (29%)	18 (31%)	23 (35%)	
Non-Hispanic or Latino	19 (56%)	45 (69%)	41 (69%)	41 (65%)	
Unknown Ethnicity	1 (3%)	1 (2%)	0 (0%)	0 (0%)	
HCV antibody seropositivity – n (%)	24 (71%)	38 (58%)	8 (14%)	3 (5%)	p < 0.0001^b,†^
HIV status – median (IQR)					
Baseline CD4 T-cell count (cells/uL)	694 (566-888)	831 (652-1031)	545 (369-913)	1008 (726-1351)	p < 0.001^a^
Baseline CD4/CD8 Ratio	0.70 (0.55-1.23)	1.66 (1.34-2.52)	0.71 (0.46-1.06)	2.16 (1.53-3.44)	p < 0.001^a^
Baseline HIV Viral Loads, RNA copies/mL*	20 (20-70)	NA	20 (20-90)	NA	NS^c^
Duration of HIV infection, months	48 (24-87)	NA	216 (126-324)	NA	p < 0.0001^c^
Duration of ART, months	36 (24-48)	NA	180 (120-264)	NA	p < 0.0001^c^

*20 = limit of detection;

NA = Not Applicable;

NS = Not Significant;

a Kruskal-Wallis;

b Chi-Square;

c Mann-Whitney Test;

† = Number of participants removed from demographic comparisons due to small cell size (Gender (n=3), Race (n=13), HCV (n=1)).

**Table 2:** Participant clinical profile, baseline urine drug screen (UDS).

Population	HIV+OP+	HIV-OP+	HIV+OP-	HIV-OP-	Comparison among groups
*N* = 221	34	65	59	63	
# of Substances – Median (IQR)	2 (2-3)	3 (2-3)	1 (0-2)	1 (0-2)	p < 0.001^a^
Reported Injection Drug Use – n (%)	17 (50%)	47 (72%)	1 (2%)	0 (0%)	p < 0.0001^b^
Opioid – n (%)					
Fentanyl	17 (50%)	54 (83%)	0 (0%)	0 (0%)	p < 0.001^c^
Buprenorphine	16 (47%)	14 (22%)	0 (0%)	0 (0%)	p < 0.05^c^
Morphine*	4 (12%)	11 (17%)	0 (0%)	0 (0%)	NS^c^
Methadone	1 (3%)	1 (2%)	0 (0%)	0 (0%)	NS^c^
Oxycodone	1 (3%)	1 (2%)	0 (0%)	0 (0%)	NS^c^
Tramadol	0 (0%)	1 (2%)	0 (0%)	0 (0%)	NS^c^
Stimulant – n (%)					
Cocaine	21 (62%)	46 (71%)	27 (46%)	19 (30%)	p < 0.0001^b^
Amphetamine	3 (9%)	4 (6%)	4 (7%)	2 (3%)	NA
Methamphetamine	2 (6%)	6 (9%)	3 (5%)	3 (5%)	NA
MDMA**	0 (0%)	6 (9%)	0 (0%)	1 (2%)	NA
Other Substances – n (%)					
Benzodiazepines	9 (29%)	8 (12%)	2 (3%)	6 (10%)	p < 0.01^b^
Cannabinoid	8 (24%)	17 (26%)	14 (24%)	25 (40%)	NS^b^
Ethyl-Glucuronide	4 (12%)	11 (18%)	12 (20%)	23 (35%)	p < 0.05^b^
Barbiturates	0 (0%)	1 (2%)	0 (0%)	0 (0%)	NA

*Also a metabolite of Heroin, **MDMA = Ecstasy,

a Kruskal-Wallis;

b Chi-Square;

c Fisher’s Exact Test (OP+ only);

NS = Not Significant;

NA = Not Applicable due to small cell size.

### 2.2 Multiparameter flow cytometry

Cryopreserved PBMCs were thawed and rested for 3 hours and analyzed by flow cytometry as previously described (8, 15). Briefly, cells were washed, resuspended in phosphate-buffered saline (PBS) with Fc receptor blocking solution (FcX), and stained using a multicolor flow panel (reagents listed in **Supplementary Table 1**). After fixation in 1% paraformaldehyde in PBS, samples were acquired on a spectral flow cytometer (Cytek Aurora) and data was analyzed by FlowJo software (v10.8.1). CD4 and CD8 T cells were gated from the Live (Aqua-) CD45+CD19-CD3+ cells and analyzed for phenotypic markers of immune activation (CD38, HLADR), immune checkpoints (CTLA-4, TIGIT, PD-1, LAG-3, TIM-3), and terminal differentiation and cellular senescence (CD28, CD57). Absolute CD4 and CD8 counts for white blood cells (WBC) were determined on a hematology analyzer (Sysmex XP-300).

### 2.3 Plasma biomarker analysis

Twenty biomarkers consisting of markers of inflammation (sTNFR-I, sTNFR-II, sCD25, TNFa, IL-6, IL-8, IL-17, IL-22, IL-1α, IL-1β, hsCRP, and D-dimer), cell adhesion (ICAM-1, VCAM-1), monocyte activation and microbial translocation (sCD14, sCD163 LBP), monocyte chemotaxis (CCL2), and anti-inflammation (IL-10), were analyzed using flow-based multiplexed bead Luminex assays as published previously (6, 16). Intestinal fatty acid binding protein (iFABP) levels were determined by ELISA (Bio-techne). For each participant (i), the expression of each plasma biomarker (j) was z-score normalized across the 20 biomarkers: z_ij_= (x_ij_ – $\bar{\text{X}}$ )/S, and a composite cytokine score was created by taking the average of 20 normalized biomarkers.

### 2.4 Statistical analysis

Participant demographics (**Table 1**) and clinical profiles (**Table 2**) were compared between four groups (Chi-Square or Kruskal-Wallis) or two groups (Fisher's Exact or Mann-Whitney U Tests) (v9.2.0 GraphPad Prism Inc). Phenotypic and plasma biomarkers were compared between groups with non-parametric Kruskal-Wallis Tests and corrected for multiple comparisons by controlling the FDR using the Benjamini and Hochberg approach (v9.2.0 GraphPad Prism Inc). Principal component analysis (PCA) on normalized plasma biomarker values (NIPALS), linear regression (base R), coefficient comparison for linear models (lavaan), negative binomial regression (MASS), and regression plots (ggeffects) were performed using R and RStudio (v.4.2.1/v.4.2.2; v.2022.07.2).

Regression models were constructed to (1) examine the relationships of HIV status and opioid use on the outcome variables, (2) control for other variables that may be important and may also differ among 4 groups in sex at birth, race, ethnicity, and age, and (3) investigate other substance use in addition to opioid use. Barbiturates could only be included in the cytokine model due to the small number of barbiturate users (n=1). In initial models, we included HIV status, opioid use (fentanyl, buprenorphine, morphine, methadone, oxycodone and tramadol), and stimulant use (cocaine, amphetamine, methamphetamine and MDMA), demographic covariates, other substance use covariates, and three two-way interactions (HIV status X opioid use, HIV status X stimulant use, opioid use X stimulant use), followed by models with pairs of these two-way interactions, followed by models with single interactions. If, at the end of this process, no interactions were significant, they were removed from the final model. If some interactions were significant, they were kept in the final models.

For analyses using linear models, in cases where coefficients for both HIV status and opioid use were significant, a near-duplicate model was conducted, but one in which the HIV status and opioid use coefficients were constrained to be equal. This constrained model was compared with the unconstrained model using an anova Chi-square difference test and the results of this test are reported. Furthermore, to determine if we could combine buprenorphine-only opioid use with non-buprenorphine opioid use into a single predictor of opioid use, we performed preliminary regression analysis with separate predictors for buprenorphine use and non-buprenorphine opioid use, followed by comparison of coefficients for non-buprenorphine with buprenorphine in constrained and unconstrained models. Because the coefficients were not significantly different (Chi-square=0.208, df=1, p=0.648), further regression models were run with all opioid use combined. Because exposure to Hepatitis C (HCV) might be expected to differ between OP+ and OP- participants, we conducted a set of preliminary regression models with HCV antibody status added to models (we did not have HCV viral load). HCV was not a significant predictor for any of our outcomes and so was dropped from the final models. Finally, to examine whether duration of HIV infection (months) and duration on ART (months), both recorded from self-report, might explain greater variation in our outcomes than HIV status alone, separate and combined models with these predictors were run. Only the outcome of cytokine score showed a relationship with duration of HIV infection or duration on ART. However, the model with HIV status, duration of HIV infection, and duration on ART was not significantly better than the model with HIV status alone (F_(195, 193)_=2.50, p=0.08); since both duration of HIV infection and duration on ART might have some recall bias and likely greater measurement error than confirmed HIV status, and the model was not significantly better, HIV status was used in final regression models.

## 3 Results

### 3.1 Study population

Characteristics of participants are shown in **Table 1**. Groups differed in some demographic characteristics such as age, race, sex, and HCV seropositivity (**Supplementary Table 2**) but these demographic characteristics were included in regression models for statistical control. As shown in **Table 1**, PWH were virally suppressed and had >500 CD4 T cell counts. The median duration of HIV infection and ART was less in the HIV+OP+ group than in the HIV+OP- group (48 *vs.* 216 months, p<0.0001; 36 *vs.* 180 months, p<0.0001, respectively). Although 95% of the participants with OUD had a known history of injection drug use (IDU), 65% self-reported active IDU at the time of their enrollment into the study (**Table 2**).

Urine drug screen (UDS) results are shown in **Table 2**. Among opioid users, Fentanyl was the most common opioid in UDS, followed by Buprenorphine, alone (27% of OP+) or in combination with other opioids (3% of OP+). Among other drugs, cocaine was most frequent with moderate to high use in all four groups; it was significantly different overall across the 4 groups (p<0.0001, **Table 2**). Cocaine use was most frequent in people with OUD, while alcohol intake was more frequent in the HIV-OP- group compared to OP+ groups (**Supplementary Table 2**).

### 3.2 Systemic inflammation is associated with opioid use regardless of HIV status

**Figure 1:**
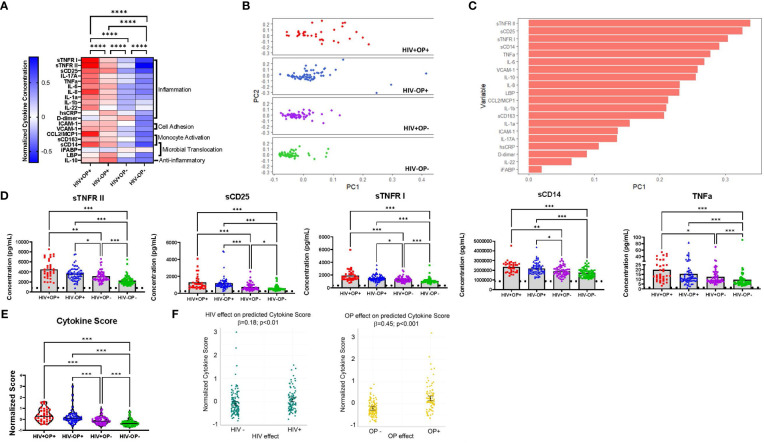
Systemic inflammation is associated with opioid use regardless of HIV status **(A)** Normalized cytokine heatmap by group (columns) and 20 cytokines (rows). Mean of normalized cytokines by participant group are displayed. **(B)** PCA of normalized cytokine values, by participant group, using non-linear iterative partial least squares (NIPALS). PC1 explains 29.9% of total variance. PC2 explains 9.9% of total variance. **(C)** Top variable loadings in PC1. **(D)** Concentration values of the top 5 cytokines by participant group. Limit of detection is indicated with a horizontal dotted line. Nonparametric Kruskal Wallis test was corrected for multiple comparisons by controlling the FDR (original FDR method of Benjamini and Hochberg). Adjusted p-values: ****p<0.0001, ***p<0.001, **p<0.01, *p<0.05. **(E)** Violin plots of cytokine scores, by participant group. Red represents HIV+OP+ (n=34), Blue represents HIV-OP+ (n=65), Purple represents HIV+OP- (n=59), and Green represents HIV-OP- (n=62). Individual plots are plotted with mean and SEM values. Nonparametric Kruskal Wallis test was corrected for multiple comparisons by controlling the FDR (original FDR method of Benjamini and Hochberg). Adjusted p-values: ****p<0.0001, ***p<0.001, **p<0.01, *p<0.05. **(F)** Scatter dot plots with mean and SEM values. Visualization of adjusted predictions of cytokine score for effects of HIV status and OP use status (while holding other covariates constant) from linear regression models (see **Table 3** for regression details).

Expression of 20 plasma biomarkers constituted predominantly of inflammatory cytokines was higher among the OP+ groups (HIV+OP+ and HIV-OP+, p<0.0001) indicating greater inflammation among people with OUD (**Figure 1A**). In an unsupervised PCA, the first principal component (PC1) explained 29.9% of the variance and better differentiated the 4 groups of participants than the second principal component (PC2), (**Figure 1B**). The HIV-OP- group had the lowest median PC1 (-0.045) while the HIV+OP+ group had the highest median PC1 (0.043). The top five components (loadings) in PC1 included sTNFR-II, sCD25, sTNFR-I, TNFa, and sCD14, which are soluble biomarkers of inflammation (**Figure 1C**). In group comparisons, the two opioid use groups had significantly greater expression of these same five biomarkers (**Figure 1D**) as well as greater expression of additional biomarkers such as IL-6, IL-10, CCL2, IL1-b, and IL-8 in both PWH and PWoH (**Supplementary Figure 4**). Additionally, the two groups with opioid use had greater expression of IL-1a in PWH and VCAM-1, sCD163, IL-17a, hsCRP, D-dimer, LBP, and ICAM-1 in PWoH (**Supplementary Figure 4**).

Group comparisons based on a composite cytokine score showed that the median cytokine score was highest among the OP+ groups (HIV+OP+, 0.38 ± 0.55; HIV-OP+, 0.21 ± 0.58), compared to the OP- groups (HIV+OP-, -0.11 ± 0.31; HIV-OP-, -0.31 ± 0.29) (**Figure 1E**). The HIV+OP+ group had the highest cytokine score, which was greater than that in the HIV+OP- (p<0.001) and HIV-OP- (p<0.001) groups. To understand whether cytokine score is affected more by HIV infection, opioid use, or other factors (including other substance use), a linear regression model was conducted (**Table 3**). Higher cytokine score was associated both with opioid use (OP+) (p<0.001) and HIV+ status (p<0.01) (**Table 3** and **Figure 1F**). In this model, the opioid effect was significantly larger than the HIV effect associated with greater expression of inflammatory cytokines (p<0.0007, Chi-squared difference test, X2 = 11.77, df=1). Demographic characteristics and other substance use were not found to be significantly associated with cytokine score (**Table 3**). There were no significant interactions between HIV infection and opioid use, HIV infection and stimulant use, or opioid use and stimulant use (and these interactions were dropped from the final model, as explained in the methods). Lacking a significant interaction suggests additive independent effects of HIV infection and opioid use in their association with higher expression of inflammatory cytokines.

**Table 3:** Linear regression results: predictors of cytokine score (n=220).

Variable	Cytokine Score Coefficient (SE)^a^
Intercept	-0.14 (0.18)
Year	-0.27 (0.06) ***
Gender (Male)	-0.09 (0.06)
Ethnicity (Hispanic)	0.03 (0.08)
Race (African American)	-0.12 (0.08)
Race (Other)	-0.13 (0.14)
Age	0.001 (0.003)
HIV Infection	0.18 (0.08) **
Opioid Use	0.44 (0.08) ***
Stimulant Use	0.05 (0.07)
Cannabinoid Use	-0.04 (0.07)
Alcohol Use	0.04 (0.08)
Benzodiazepine Use	-0.11 (0.09)
Barbiturate Use	-0.05 (0.43)
Smoking	0.07 (0.07)
R Squared	0.32
F-statistic	8.315 on 14 and 199
P-value	<5.8e-14***

a p-values: ***p<0.001, **p<0.01.

### 3.3 Immune activation (HLADR+CD38+) and PD1 expression on CD8 T cells are associated with HIV and opioid use

**Figure 2:**
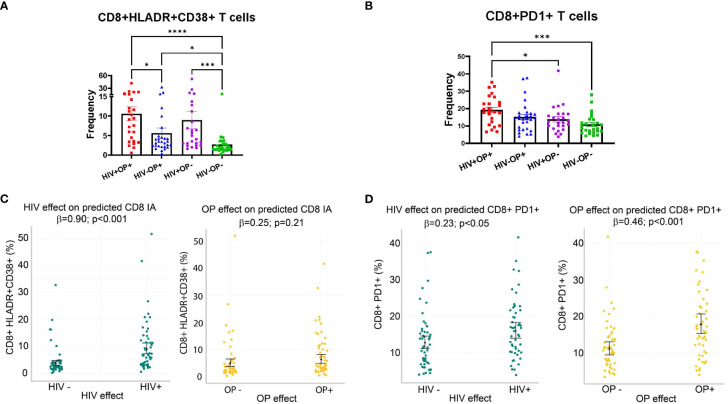
Immune activation (HLADR+CD38+) and PD1 expression on CD8 T cells are increased in populations with HIV and opioid use. **(A, B)** Scatter dot plots with bars, mean and SEM values for frequencies of HLADR+CD38+ **(A)** and PD1+ **(B)** in CD8 T cells. Red represents HIV+OP+ (n=25), Blue represents HIV-P+ (n=29), Purple represents HIV+OP- (n=25), and Green represents HIV-OP- (n=26). Nonparametric Kruskal Wallis test was corrected for multiple comparisons by controlling the FDR (original FDR method of Benjamini and Hochberg). Adjusted p-values: ****p<0.0001, ***p<0.001, *p<0.05. **(C, D)** Scatter dot plots with mean and SEM values. Visualization of adjusted predictions for effects of HIV status and OP use status (while holding other covariates constant) from negative binomial regression models used to predict HLADR+CD38+ **(C)** and PD1 **(D)** in CD8 T cells (see **Table 4** for regression details).

T cell IA was analyzed based on the dual expression of HLA-DR and CD38 on CD4 and CD8 T cells (**Supplemental Figure 2**). Group comparisons based on CD8 T cell IA showed significant differences among groups (**Figure 2A**). Compared to the HIV-OP- group (2.7% ± 3.0), CD8 T cell IA was significantly greater in all other groups; the HIV+OP+ group had the highest expression (10.6% ± 8.9, p<0.0001), followed by the HIV+OP- group (8.9% ± 11, p<0.001) and HIV-OP+ group (5.6% ± 6.9, p<0.05). CD8 T cell IA was greater in the HIV+OP+ group compared to the HIV-OP+ group (p<0.05). Expression of PD1, considered to be a marker of immune activation, was highest in CD8 T cells of the HIV+OP+ group (19.2% ± 8.3) as compared to the HIV+OP- (13.8% ± 7.7, p<0.05) and HIV-OP- (10.88% ± 5.9, p<0.001) groups (**Figure 2B**). Frequencies of other checkpoint markers of exhaustion (TIM-3, LAG-3, CTLA-4, TIGIT) were not significantly different among the groups in CD4 and CD8 T cells (data not shown).

To understand whether IA is affected more by HIV infection, opioid use, or other substance use, a negative binomial regression model was conducted, controlling for other factors (including other types of substance use) (**Table 4**). HIV+ status, but not opioid use (OP+), was associated with higher expression of CD8 HLADR+CD38+ (p<0.001, **Table 4** and **Figure 2C**). Higher PD1 expression on CD8 T cells was associated with both HIV+ status (p<0.05) and opioid use (OP+) (p<0.001) (**Table 4** and **Figure 2D**). There were no significant interactions between HIV infection and opioid use, HIV infection and stimulant use, or opioid use and stimulant use in any of these models (and these interactions were dropped from the final model, as explained in the methods).

**Table 4:** Negative binomial regression results: predictors of immune activation and senescence in CD4 and CD8 T cells.

Variable	Immune Activation^a^ Coefficient (SE) ^b^				Immune Senescence^a^ Coefficient (SE) ^b^			
	CD4 HLADR+CD38+	CD8 HLADR+CD38+	CD4 PD1+	CD8 PD1+	CD4 CD28-CD57+	CD8 CD28-CD57+	CD4 CD28-CD57+CD38+	CD8 CD28-CD57+CD38+
Intercept	-5.17 (0.61) ***	-4.00 (0.57) ***	-2.42 (0.30) ***	-3.01 (0.32) ***	-5.11 (0.84) ***	-1.76 (0.33) ***	-2.30 (0.71) **	-2.96 (0.50) ***
Gender (Male)	-0.02 (0.19)	-0.04 (0.18)	0.03 (0.09)	0.07 (0.10)	0.18 (0.27)	0.05 (0.11)	0.25 (0.23)	-0.10 (0.16)
Ethnicity (Hispanic)	0.18 (0.21)	0.11 (0.20)	-0.16 (0.11)	-0.09 (0.12)	0.07 (0.31)	0.01 (0.12)	-0.13 (0.26)	-0.01 (0.18)
Race (African American)	0.37 (0.23)	0.16 (0.22)	0.18 (0.12)	-0.01 (0.13)	0.74 (0.33) *	0.21 (0.13)	-0.18 (0.28)	0.18 (0.20)
Race (Other)	0.31 (0.36)	0.03 (0.37)	0.53 (0.20) **	-0.04 (0.22)	1.37 (0.54) *	0.27 (0.23)	-0.08 (0.50)	-0.08 (0.35)
Age	0.01 (0.01)	-0.01 (0.01)	0.01 (0.01) **	0.01 (0.01) *	0.04 (0.01) *	0.01 (0.01)	-0.01 (0.01)	0.01 (0.01)
HIV Infection	0.41 (0.18) *	0.90 (0.17) ***	0.18 (0.09) *	0.23 (0.10) *	0.16 (0.26)	0.12 (0.10)	0.46 (0.22) *	0.50 (0.15) **
Opioid Use	0.19 (0.22)	0.25 (0.21)	0.06 (0.11)	0.46 (0.12) ***	-0.03 (0.32)	0.15 (0.13)	0.89 (0.27) **	1.22 (0.20) ***
Stimulant Use	0.52 (0.20) **	0.90 (0.19) ***	0.24 (0.10) *	0.08 (0.11)	0.77 (0.28) **	0.17 (0.11)	0.06 (0.24)	0.48 (0.17) **
Cannabinoid Use	-0.13 (0.19)	-0.12 (0.18)	-0.08 (0.10)	-0.05 (0.10)	-0.21 (0.27)	0.05 (0.11)	-0.12 (0.23)	-0.29 (0.16)
Alcohol Use	-0.22 (0.22)	0.14 (0.20)	0.01 (0.11)	0.18 (0.12)	-0.39 (0.31)	-0.10 (0.12)	0.10 (0.26)	0.17 (0.19)
Benzodiazepine Use	-0.40 (0.30)	-0.15 (0.26)	-0.14 (0.14)	-0.14 (0.15)	-0.29 (0.41)	0.01 (0.16)	-0.42 (0.34)	0.08 (0.24)
Smoking	-0.12 (0.25)	0.31 (0.25)	-0.31 (0.12) *	-0.002 (0.14)	-1.07 (0.34) **	-0.25 (0.14)	-0.20 (0.30)	-0.18 (0.21)

a n=103 for each regression,

b p-values: ***p<0.001, **p<0.01, *p<0.05.

In group comparisons, IA and PD1 expression in CD4 T cells were not different between the 4 groups (**Supplemental Figure 3A, B**). However, negative binomial regression models, controlling for other factors, indicated that higher expression levels of CD4 IA (HLADR+CD38+) and PD1 were associated with HIV+ status (p<0.05), but not opioid use (OP+) (**Table 4** and **Supplementary Figures 3C, D**). Stimulant use was associated with increased IA (HLADR+CD38+) and PD1 expression in CD4 T cells and IA HLADR+CD38+) in CD8 T cells compared to non-stimulant use (**Table 4**). There were no significant interactions between HIV infection and opioid use, HIV infection and stimulant use, or opioid use and stimulant use in any of these models (and these interactions were dropped from the final model, as explained in the methods).

### 3.4 CD38 expressing terminally differentiated senescent-like T cells are associated with opioid use

**Figure 3:**
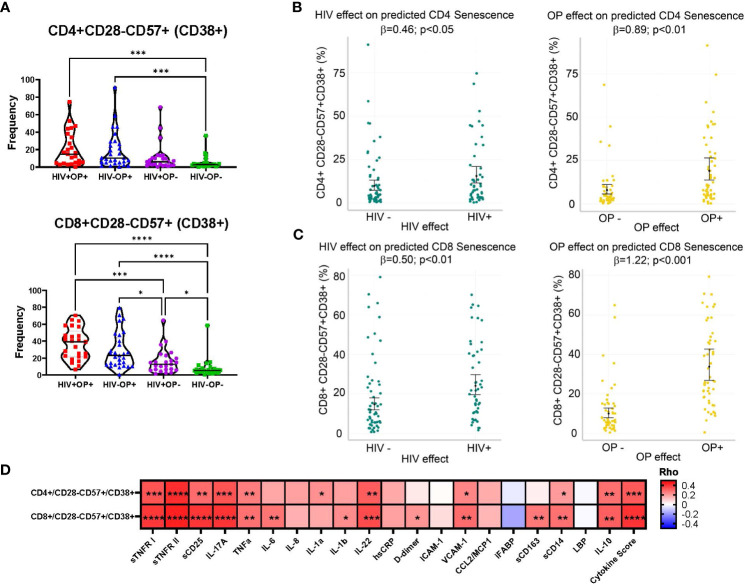
CD38 expressing terminally differentiated senescent-like T cells are highly expressed in populations with opioid use. **(A)** Violin plots by participant group with mean and SEM confidence intervals for frequencies of CD28-CD57+CD38+ in CD4 and CD8 T cells. Nonparametric Kruskal Wallis test was corrected for multiple comparisons by controlling the FDR (original FDR method of Benjamini and Hochberg). Red represents HIV+OP+ (n=25), Blue represents HIV-OP+ (n=29), Purple represents HIV+OP- (n=25), Green represents HIV-OP- (n=26). Adjusted p-values: ****p<0.0001, ***p<0.001, **p<0.01, *p<0.05. **(B, C)** Scatter dot plots with mean and SEM values. Visualization of adjusted predictions for effects of HIV status and OP use status (while holding other covariates constant) from negative binomial regression models used to predict CD28-CD57+CD38+ in CD4 **(B)** and CD8 **(C)** in T cells (see **Table 4** for regression details). **(D)** Spearman correlation matrix of 20 individual normalized cytokines and normalized cytokine score with CD28-CD57+CD38+ expression on CD4 and CD8 T cells. Adjusted p-values: ****p<0.0001, ***p<0.001, **p<0.01, *p<0.05, indicate significant correlation between two variables in the box.

Since chronic HIV infection is associated with premature cellular senescence (17), we examined the frequencies of senescent-like phenotype (CD28-CD57+) and CD38 (18) on T cells. Although CD28-CD57+ subsets in CD4 and CD8 T cells did not differ between groups (data not shown), CD38 expression on CD28-CD57+ CD4 T cells was higher in both OP+ groups (p<0.001) compared to HIV-OP- (**Figure 3A**). On CD8 T cells, CD38 expression on CD28-CD57+ was higher in both OP+ groups (p<0.0001) and HIV+OP- (p<0.05) compared to HIV-OP- as well as HIV+OP+ (p<0.001) compared to HIV+OP-. In negative binomial regression models, controlling for other factors, both HIV+ status and opioid use (OP+) were associated with significantly greater CD38+ expression on CD28-CD57+ cells in CD4 (p<0.05 and p<0.01, respectively, **Table 4** and **Figure 3B**) as well as CD8 T cells (p<0.01 and p<0.001, respectively, **Table 4** and **Figure 3C**). There were no significant interactions between HIV infection and opioid use, HIV infection and stimulant use, or opioid use and stimulant use in CD38 expression on CD28-CD57+ T cells (and these interactions were dropped from the final model, as explained in the methods). In both CD4 and CD8 T cell compartments, the cellular phenotype CD28-CD57+CD38+, was significantly positively associated with cytokine score and biomarkers such as sTNFR-I, sTNFR-II, sCD25, IL-17, TNFα, IL-6, IL-22, sCD14 (**Figure 3D**).

## 4 Discussion

Excessive inflammation and immune activation are known to be prevalent in PWH and have been linked to higher risk for comorbidities (19, 20). Systemic inflammation is also known to be associated with substance use and addictive disorders that could impact the progression of diseases (9). Our research team was uniquely positioned to enroll people with and without OUD among both PWH and PWoH in this study. Furthermore, we used rigorous statistical methods, taking into consideration demographic differences, polydrug use and strict confirmation of opioid use, for our immunological studies. We have shown that opioid use was independently associated with higher inflammation while only HIV status independently contributed to cellular immune activation. Our OUD cohort specifically mirrors the trend of synthetic opioid usage with a majority of those using opioids testing positive for potent fentanyl and analogues in the study (whether intentionally or unintentionally). This study is timely in its unique examination of the immune response to fentanyl use, the third wave of the overdose crisis, as well as fentanyl and stimulant co-use, the deadly fourth wave (21–24). The first two waves consisted of prescription opioids and heroin, respectively. It is possible that the use of fentanyl is a reflection of the nature of the current drug supply available on the streets. Stimulant use has previously been shown to be associated with significant increase in T cell immune activation (25). While controlling for HIV infection and opioid use, stimulant use was associated with greater immune activation. Our models will need to control for new entrants into the opioid epidemic such as Xylazine in the future (26).

Excessive T cell immune activation in virally suppressed PWH and its role in HIV disease progression are well known in the literature (6, 20, 27). One study reported that PWH with history of IDU and OUD, who were on oral methadone treatment, had greater levels of inflammation and immune activation than PWH with no history of OUD (11, 28). Our study participants with OUD involved predominantly IDU with self-reported consistent opioid use for >90 days until the day of study enrollment and a positive opioid test in the UDS preceding the blood draw which was done on the enrollment day. In our cohort, higher CD8 T cell immune activation in people with OUD was only found to have a significant HIV effect (except for CD8 PD1+). While *in vivo* activation of CD4+ T cells is more dependent on the specificities for persistence of antigens, CD8+ T cells can be activated by inflammatory cytokines, independent of Ag specificity (29–31). The apparent difference seen with CD4 and CD8 immune activation in our study might be explained by the differential responsiveness of CD4+ and CD8+ T cells toward inflammatory cytokines. Higher CD8 T cell activation found in HIV+OP+ could reflect their response to inflammation.

Using the UDS, we also noted the high frequency of polydrug use, particularly cocaine among stimulant use, among participants in all 4 groups. We found that stimulant use (unlike opioid use, except for CD8 PD1+) was independently associated with an increase in CD4 and CD8 T cell- immune activation. These observations suggest that stimulants and opioids may have different interactions with immune cells. Additional studies are necessary to fully understand the downstream effects of polydrug use on immunity, to better design treatments for people with OUD.

Our results aligned with previous reports examining the state of inflammation in virally suppressed PWH with IDU and OUD (9, 28, 32, 33), showing increased expression of IL-6 (opioid use) (32), IL-8, sTNFR-II (methadone use) (28), and LBP, hsCRP, sTNFR-I, sTNFR-II, sCD14, sCD163 (heroin use) (28, 32, 33). In our study, plasma biomarkers were selected based on their reported association with inflammation (sTNFR-II, sTNFR-I, sCD25, TNFa, IL-6, IL-8, IL-1a, IL-1b, IL17A (Th17), IL-22 (Th17)) (34), cardiovascular disease (ICAM-I, VCAM-I, hsCRP), co-morbidities (IL-6, VCAM-I, ICAM-I, sTNFR-II, and sTNFR-I), monocyte activation and microbial translocation (sCD14, sCD163, iFABP, D-dimer, LBP) (32), HIV disease progression (MCP1/CCL2, sCD14) and mortality (sCD14) in the context of HIV. We probed new biomarkers that have not been reported (sCD25, VCAM-I, ICAM-I, CCL2) that provide additional insight into the systemic inflammation profile with fentanyl use and HIV infection.

Based on regression modeling, an opioid effect was greater than an HIV effect on inflammation. The route of injection drug use can also impact systemic inflammation. Compared to oral use, the act of drug injection and penetration of the epidermal barrier may constitute exposures that can cause infections, exacerbating inflammation (35). Our results show that HIV infection and chronic opioid use are each independently associated with systemic inflammation but chronic opioid use is further associated with systemic inflammation among PWH, with HIV+OP+ having the highest systemic inflammation. Further investigation is warranted to find the potential link between inflammation and their clinical significance among people who use fentanyl.

In PWoH, opioid use was associated with higher expression of plasma biomarkers VCAM-1, sCD163, IL-17a, hsCRP, D-dimer, LBP, and ICAM-1. Studies of OUD, without HIV infection, have shown increased plasma inflammatory cytokines levels (TNF-α, CRP, IL-8, IL-6, and BDNF) (36). Our extensive cytokine panel has broadened what is currently known in the field. Although we saw a higher expression of the anti-inflammatory molecule IL-10 with opioid use, this cytokine may reflect a response to inflammation through negative feedback regulation that affects the control and resolution of inflammation.

Chronic HIV infection is associated with premature cellular senescence, especially in the context of aging as shown by decreased cell density, increased levels of p16, and decreased telomere length (37). Concurrent substance use disorder in PWH may contribute to accelerated aging by increasing persistent inflammation (37). In the context of OUD and HIV, our study specifically examined cellular phenotypic features and plasma biomarkers of immune senescence. CD38 is an immunomodulatory molecule, expressed on multiple immune cell types, and is involved with different functions such as inflammation, cellular migration, phagocytosis, antigen presentation, and NAD+ metabolism during inflammation (38). Increased expression of CD38 is associated with aging and senescence, resulting in age-related NAD decline and mitochondrial dysfunction (39). There is also an association between inflammation and CD38 in association with NAD decline (40). In regression modeling, we demonstrated no significant interactions between HIV infection and opioid use, suggesting additive independent effects of HIV and opioid use associated with the expression of CD38 on senescent-like T cells. Overall, CD38 expression on terminally differentiated senescent-like T cells was positively associated with composite cytokine score and individual biomarkers such as sTNFR-I, sTNFR-II, sCD25, IL-17, TNFα, IL-6, IL-22, and sCD14, suggesting that the accumulation of senescent cells with secretion associated senescent phenotype (SASP) (41) could be a major cause of ongoing inflammation.

A limitation of our study was the lower number of recruited PWH with OUD (HIV+OP+) in comparison to the other groups. At the Syringe Services Program, about 42% of the participants are victims of structural inequities and social determinants of health such as homelessness (42), leading to unique challenges to recruitment that have been previously reported (43). Specifically, at an individual level, challenges included language barriers, transportation issues, and profound mistrust of medical research and vaccines (43–45). It was also challenging to recruit virally suppressed PWH on ART which was an inclusion criterion and contributed to the lower numbers and shorter duration of ART use in HIV+OP+ versus HIV+ OP- participants. However, controlling for ongoing viremia is also a strength of our study as it dissociates the effects of replicating virus on immune measures. In our cohort, we investigated for presence of antibodies against HCV, a common viral infection associated with a history of injection drug use, and previous studies have linked HCV viremic PWH and IDU with elevated levels of immune activation (46). In regression modeling, HCV antibody status did not have a significant effect on inflammation, immune activation, and senescence in our cohort (data not shown) but we did not have data on active HCV infection.

Another limitation is the potential heterogeneity introduced by concurrent substance use, e.g. stimulants (22) in a study aiming to investigate effect of opioids. This is, however, the reality of substance use, and including data of additional substances in people who were predominantly opioid users adds to the clinical usefulness of our findings (21–24). To mitigate the effects of this heterogeneity, we carefully measured the use of other substances and statistically controlled for each additional substance used. This approach enabled us to show the immune effects of stimulant use (the most prevalent other substance use) independent of opioid use. Despite noted limitations, our stringent statistical approaches were able to identify the link between inflammation, immune activation, and chronic opioid use in virally suppressed PWH which has relevance to HIV disease progression and non-AIDS comorbidities as well as to future interventions toward permanent HIV remission. Our study warrants a better mechanistic understanding of how the syndemic of HIV and OUD and OUD by itself alters immune status to provide insight for developing new approaches to improve the health outcomes in this population.

## Author’s note

This article was presented in part as an oral presentation at the 24th International AIDS Conference, Montreal, CA, July 31, 2022. Session People at the Centre. Abstract B58.

## Data availability statement

The original contributions presented in the study are included in the article/**Supplementary Material**. Further inquiries can be directed to the corresponding author.

## Ethics statement

The studies involving humans were approved by University of Miami Institutional Review Board (University of Miami IRB # 20200178). The studies were conducted in accordance with the local legislation and institutional requirements. The participants provided their written informed consent to participate in this study.

## Author contributions

CMD: Data curation, Formal Analysis, Methodology, Validation, Visualization, Writing – original draft, Writing – review & editing, Investigation. CMN: Data curation, Formal Analysis, Software, Validation, Visualization, Writing – review & editing. DJF: Supervision, Writing – review & editing, Methodology, Validation. AK: Data curation, Formal Analysis, Software, Validation, Visualization, Writing – review & editing. DWF: Methodology, Supervision, Writing – review & editing. NN: Methodology, Writing – review & editing. AI: Formal Analysis, Software, Visualization, Writing – review & editing. PPG: Formal Analysis, Visualization, Writing – review & editing. DTJ: Methodology, Supervision, Writing – review & editing. AER: Methodology, Supervision, Writing – review & editing. RNP: Methodology, Supervision, Writing – review & editing, Investigation, Visualization. HET: Methodology, Supervision, Writing – review & editing. SP: Conceptualization, Investigation, Methodology, Supervision, Validation, Visualization, Writing – review & editing. SGP: Conceptualization, Funding acquisition, Investigation, Methodology, Resources, Supervision, Validation, Visualization, Writing – review & editing.

## Supplementary material

Supplementary Figure 1 Opioid Immunity Study (OPIS) Study Design. **(A)** Four groups are recruited based on HIV status and opioid use status. Urine samples are collected to test opioid and polydrug use. Red represents HIV+OP+, Blue represents HIV-OP+, Purple represents HIV+OP-, and Green represents HIV-OP-. Participants of all genders are recruited into the study. **(B)** Blood samples are collected and processed to collect plasma and peripheral blood mononuclear cells to analyze inflammation and immune activation. Design Figure was created with BioRender.com.

Supplementary Figure 2 Gating strategy used to define HLADR+CD38+ within CD4 and CD8 T cells population of peripheral blood mononuclear cells. Doublets were excluded. Representative plots from an HIV+OP+ individual.

Supplementary Figure 3 Immune activation (HLADR+CD38+) and PD1 expression on CD4 T cells are increased in populations with HIV and opioid use. **(A, B)** Scatter dot plots with bars, mean and SEM values for frequencies of HLADR+CD38+ **(A)** and PD1+ **(B)** in CD4 T cells. Red represents Gp1 (HIV+OP+, n=25), Blue represents Gp2 (HIV-OP+, n=29), Purple represents Gp3 (HIV+OP-, n=25), Green represents Gp4 (HIV-OP-, n=26). Nonparametric Kruskal Wallis test was corrected for multiple comparisons by controlling the FDR (original FDR method of Benjamini and Hochberg). **(C, D)** Scatter dot plots with mean and SEM values. Visualization of adjusted predictions for effects of HIV status and OP use (while holding other covariates constant) from negative binomial regression models for predicted HLADR+CD38+ **(C)** and PD1 **(D)** in CD4 T cells (see **Table 4** for regression details).

Supplementary Figure 4 Concentrations of inflammatory biomarkers. Scatter dot plots with bars by group with mean and SEM confidence intervals of 20 non-normalized cytokines. Red represents HIV+OP+ (n=34), Blue represents HIV-OP+ (n=65), Purple represents HIV+OP- (n=59), and Green represents HIV-OP- (n=62). Nonparametric Kruskal Wallis test was corrected for multiple comparisons by controlling the FDR (original FDR method of Benjamini and Hochberg). Adjusted p-values: ****p<0.0001, ***p<0.001, **p<0.01, *p<0.05. The limit of detection is indicated with a dotted line.

Supplementary Table 1 Panel of surface markers and reagents used in multiparameter flow cytometry.

Supplementary Table 2 Multiple group comparisons of significant variables in cohort demographics and participant clinical profile, baseline urine drug screen (UDS).

## References

[1] Levitt A Mermin J Jones CM See I Butler JC. Infectious diseases and injection drug use: public health burden and response. J Infect Dis (2020) 222(Suppl 5):S213–S7. doi: 10.1093/infdis/jiaa432 32877539

[2] Hodder SL Feinberg J Strathdee SA Shoptaw S Altice FL Ortenzio L. The opioid crisis and HIV in the USA: deadly synergies. Lancet (2021) 397(10279):1139–50. doi: 10.1016/S0140-6736(21)00391-3 33617769

[3] (NIDA) NIoDA. Part 3: The Connection between Substance Use Disorders and HIV. (2021). Bethesda: National Institute on Drug Abuse.

[4] Volkow ND Baler RD Normand JL. The unrealized potential of addiction science in curbing the HIV epidemic. Curr HIV Res (2011) 9(6):393–5. doi: 10.2174/157016211798038605 PMC3520050 21999774

[5] Degenhardt L Whiteford HA Ferrari AJ Baxter AJ Charlson FJ Hall WD. Global burden of disease attributable to illicit drug use and dependence: findings from the Global Burden of Disease Study 2010. Lancet (2013) 382(9904):1564–74. doi: 10.1016/S0140-6736(13)61530-5 23993281

[6] de Armas LR Pallikkuth S George V Rinaldi S Pahwa R Arheart KL. Reevaluation of immune activation in the era of cART and an aging HIV-infected population. JCI Insight (2017) 2(20):e95726. doi: 10.1172/jci.insight.95726 29046481 PMC5846952

[7] de Armas LR Pallikkuth S Pan L Rinaldi S Cotugno N Andrews S. Single Cell Profiling Reveals PTEN Overexpression in Influenza-Specific B cells in Aging HIV-infected individuals on Anti-retroviral Therapy. Sci Rep (2019) 9(1):2482. doi: 10.1038/s41598-019-38906-y 30792481 PMC6385500

[8] Pallikkuth S de Armas LR Rinaldi S George VK Pan L Arheart KL. Dysfunctional peripheral T follicular helper cells dominate in people with impaired influenza vaccine responses: Results from the FLORAH study. PloS Biol (2019) 17(5):e3000257. doi: 10.1371/journal.pbio.3000257 31100059 PMC6542545

[9] Morcuende A Navarrete F Nieto E Manzanares J Femenia T. Inflammatory biomarkers in addictive disorders. Biomolecules (2021) 11(12):1824. doi: 10.3390/biom11121824 34944470 PMC8699452

[10] Doggui R Elsawy W Conti AA Baldacchino A. Association between chronic psychoactive substances use and systemic inflammation: A systematic review and meta-analysis. Neurosci Biobehav Rev (2021) 125:208–20. doi: 10.1016/j.neubiorev.2021.02.031 33639179

[11] Azzoni L Metzger D Montaner LJ. Effect of opioid use on immune activation and HIV persistence on ART. J Neuroimmune Pharmacol (2020) 15(4):643–57. doi: 10.1007/s11481-020-09959-y PMC7719088 32974750

[12] Bartholomew TS Feaster DJ Patel H Forrest DW Tookes HE. Reduction in injection risk behaviors after implementation of a syringe services program, Miami, Florida. J Subst Abuse Treat (2021) 127:108344. doi: 10.1016/j.jsat.2021.108344 34134863 PMC8221088

[13] Harris PA Taylor R Minor BL Elliott V Fernandez M O'Neal L. The REDCap consortium: Building an international community of software platform partners. J BioMed Inform (2019) 95:103208. doi: 10.1016/j.jbi.2019.103208 31078660 PMC7254481

[14] Pallikkuth S Parmigiani A Silva SY George VK Fischl M Pahwa R. Impaired peripheral blood T-follicular helper cell function in HIV-infected nonresponders to the 2009 H1N1/09 vaccine. Blood (2012) 120(5):985–93. doi: 10.1182/blood-2011-12-396648 PMC3412336 22692510

[15] Rinaldi S de Armas L Dominguez-Rodriguez S Pallikkuth S Dinh V Pan L. T cell immune discriminants of HIV reservoir size in a pediatric cohort of perinatally infected individuals. PloS Pathog (2021) 17(4):e1009533. doi: 10.1371/journal.ppat.1009533 33901266 PMC8112655

[16] de Armas LR Pallikkuth S Pan L Rinaldi S Pahwa R Pahwa S. Immunological age prediction in HIV-infected, ART-treated individuals. Aging (Albany NY) (2021) 13(19):22772–91. doi: 10.18632/aging.203625 PMC8544329 34635604

[17] Banica L Vlaicu O Jipa R Abagiu A Nicolae I Neaga E. Exhaustion and senescence of CD4 and CD8 T cells that express co-stimulatory molecules CD27 and CD28 in subjects that acquired HIV by drug use or by sexual route. Germs (2021) 11(1):66–77. doi: 10.18683/germs.2021.1242 33898343 PMC8057851

[18] Chou JP Ramirez CM Wu JE Effros RB. Accelerated aging in HIV/AIDS: novel biomarkers of senescent human CD8+ T cells. PloS One (2013) 8(5):e64702. doi: 10.1371/journal.pone.0064702 23717651 PMC3661524

[19] Leeansyah E Malone DF Anthony DD Sandberg JK. Soluble biomarkers of HIV transmission, disease progression and comorbidities. Curr Opin HIV AIDS (2013) 8(2):117–24. doi: 10.1097/COH.0b013e32835c7134 23274365

[20] Alcaide ML Parmigiani A Pallikkuth S Roach M Freguja R Della Negra M. Immune activation in HIV-infected aging women on antiretrovirals–implications for age-associated comorbidities: a cross-sectional pilot study. PloS One (2013) 8(5):e63804. doi: 10.1371/journal.pone.0063804 23724003 PMC3665816

[21] Wilson N Kariisa M Seth P Smith HT Davis NL. Drug and opioid-involved overdose deaths - United States, 2017-2018. MMWR Morb Mortal Wkly Rep (2020) 69(11):290–7. doi: 10.15585/mmwr.mm6911a4 PMC7739981 32191688

[22] Kariisa M Scholl L Wilson N Seth P Hoots B. Drug overdose deaths involving cocaine and psychostimulants with abuse potential - United States, 2003-2017. MMWR Morb Mortal Wkly Rep (2019) 68(17):388–95. doi: 10.15585/mmwr.mm6817a3 PMC6541315 31048676

[23] Spencer MR Warner M Bastian BA Trinidad JP Hedegaard H. Drug overdose deaths involving fentanyl, 2011-2016. Natl Vital Stat Rep (2019) 68(3):1–19. 31112123

[24] Fogger SA. Methamphetamine use: A new wave in the opioid crisis? J Addict Nurs (2019) 30(3):219–23. doi: 10.1097/JAN.0000000000000298 31478970

[25] Kim SG Jung JB Dixit D Rovner R Jr. Zack JA Baldwin GC. Cocaine exposure enhances permissiveness of quiescent T cells to HIV infection. J Leukoc Biol (2013) 94(4):835–43. doi: 10.1189/jlb.1112566 PMC3774841 23817564

[26] DEA. The Growing Threat of Xylazine and its Mixture with Illicit Drugs. (2022). Washington, DC: US Department of Justice, Drug Enfo.

[27] Erlandson KM Ng DK Jacobson LP Margolick JB Dobs AS Palella FJ Jr.. Inflammation, immune activation, immunosenescence, and hormonal biomarkers in the frailty-related phenotype of men with or at risk for HIV infection. J Infect Dis (2017) 215(2):228–37. doi: 10.1093/infdis/jiw523 PMC5897840 27799351

[28] Azzoni L Giron LB Vadrevu S Zhao L Lalley-Chareczko L Hiserodt E. Methadone use is associated with increased levels of sCD14, immune activation, and inflammation during suppressed HIV infection. J Leukoc Biol (2022) 112(4):733-44. doi: 10.1002/JLB.4A1221-678RR PMC13730277 35916053

[29] Bastidas S Graw F Smith MZ Kuster H Gunthard HF Oxenius A. CD8+ T cells are activated in an antigen-independent manner in HIV-infected individuals. J Immunol (2014) 192(4):1732–44. doi: 10.4049/jimmunol.1302027 24446519

[30] Catalfamo M Le Saout C Lane HC. The role of cytokines in the pathogenesis and treatment of HIV infection. Cytokine Growth Factor Rev (2012) 23(4-5):207–14. doi: 10.1016/j.cytogfr.2012.05.007 PMC3726258 22738931

[31] Cox MA Kahan SM Zajac AJ. Anti-viral CD8 T cells and the cytokines that they love. Virology (2013) 435(1):157–69. doi: 10.1016/j.virol.2012.09.012 PMC3580945 23217625

[32] Kholodnaia A So-Armah K Cheng D Gnatienko N Patts G Samet JH. Impact of illicit opioid use on markers of monocyte activation and systemic inflammation in people living with HIV. PloS One (2022) 17(5):e0265504. doi: 10.1371/journal.pone.0265504 35511802 PMC9070930

[33] Hileman CO Bowman ER Gabriel J Kettelhut A Labbato D Smith C. Impact of heroin and HIV on gut integrity and immune activation. J Acquir Immune Defic Syndr (2022) 89(5):519–26. doi: 10.1097/QAI.0000000000002893 PMC8901022 35001040

[34] Babu H Ambikan AT Gabriel EE Svensson Akusjarvi S Palaniappan AN Sundaraj V. Systemic inflammation and the increased risk of inflamm-aging and age-associated diseases in people living with HIV on long term suppressive antiretroviral therapy. Front Immunol (2019) 10:1965. doi: 10.3389/fimmu.2019.01965 31507593 PMC6718454

[35] Hrycko A Mateu-Gelabert P Ciervo C Linn-Walton R Eckhardt B. Severe bacterial infections in people who inject drugs: the role of injection-related tissue damage. Harm Reduct J (2022) 19(1):41. doi: 10.1186/s12954-022-00624-6 35501854 PMC9063270

[36] Wang TY Lu RB Lee SY Chang YH Chen SL Tsai TY. Association between inflammatory cytokines, executive function, and substance use in patients with opioid use disorder and amphetamine-type stimulants use disorder. Int J Neuropsychopharmacol (2023) 26(1):42–51. doi: 10.1093/ijnp/pyac069 36181736 PMC9850661

[37] Cohen J Torres C. HIV-associated cellular senescence: A contributor to accelerated aging. Ageing Res Rev (2017) 36:117–24. doi: 10.1016/j.arr.2016.12.004 PMC5584608 28017881

[38] Hogan KA Chini CCS Chini EN. The multi-faceted ecto-enzyme CD38: roles in immunomodulation, cancer, aging, and metabolic diseases. Front Immunol (2019) 10:1187. doi: 10.3389/fimmu.2019.01187 31214171 PMC6555258

[39] Camacho-Pereira J Tarrago MG Chini CCS Nin V Escande C Warner GM. CD38 dictates age-related NAD decline and mitochondrial dysfunction through an SIRT3-dependent mechanism. Cell Metab (2016) 23(6):1127–39. doi: 10.1016/j.cmet.2016.05.006 PMC4911708 27304511

[40] Zeidler JD Hogan KA Agorrody G Peclat TR Kashyap S Kanamori KS. The CD38 glycohydrolase and the NAD sink: implications for pathological conditions. Am J Physiol Cell Physiol (2022) 322(3):C521–C45. doi: 10.1152/ajpcell.00451.2021 PMC8917930 35138178

[41] Gasek NS Kuchel GA Kirkland JL Xu M. Strategies for targeting senescent cells in human disease. Nat Aging (2021) 1(10):870–9. doi: 10.1038/s43587-021-00121-8 PMC8612694 34841261

[42] Iyengar S Kravietz A Bartholomew TS Forrest D Tookes HE. Baseline differences in characteristics and risk behaviors among people who inject drugs by syringe exchange program modality: an analysis of the Miami IDEA syringe exchange. Harm Reduct J (2019) 16(1):7. doi: 10.1186/s12954-019-0280-z 30674334 PMC6343273

[43] Batista P Deren S Banfield A Silva E Cruz M Garnes P. Challenges in recruiting people who use drugs for HIV-related biomedical research: perspectives from the field. AIDS Patient Care STDS (2016) 30(8):379–84. doi: 10.1089/apc.2016.0135 PMC4991596 27509238

[44] Strathdee SA Abramovitz D Harvey-Vera A Vera CF Rangel G Artamonova I. Correlates of coronavirus disease 2019 (COVID-19) vaccine hesitancy among people who inject drugs in the San Diego-Tijuana border region. Clin Infect Dis (2022) 75(1):e726–e33. doi: 10.1093/cid/ciab975 PMC8690110 35024825

[45] Cepeda JA Feder KA Astemborski J Schluth C Kirk GD Mehta SH. COVID-19 vaccine hesitancy and vaccination status in a community-based cohort of people who inject drugs in Baltimore, Maryland, March-June 2021. Public Health Rep (2022) 137(5):1031–40. doi: 10.1177/00333549221110299 PMC9357826 35848111

[46] Markowitz M Deren S Cleland C La Mar M Silva E Batista P. Chronic hepatitis C virus infection and the proinflammatory effects of injection drug use. J Infect Dis (2016) 214(9):1376–82. doi: 10.1093/infdis/jiw373 PMC5079368 27521361

