# Supplementary figures and images for: Opioids exacerbate inflammation in people with well-controlled HIV
Source: Front Immunol. 2023 Nov 1;14:1277491. doi: 10.3389/fimmu.2023.1277491 (PMC10646416; doi:10.3389/fimmu.2023.1277491)

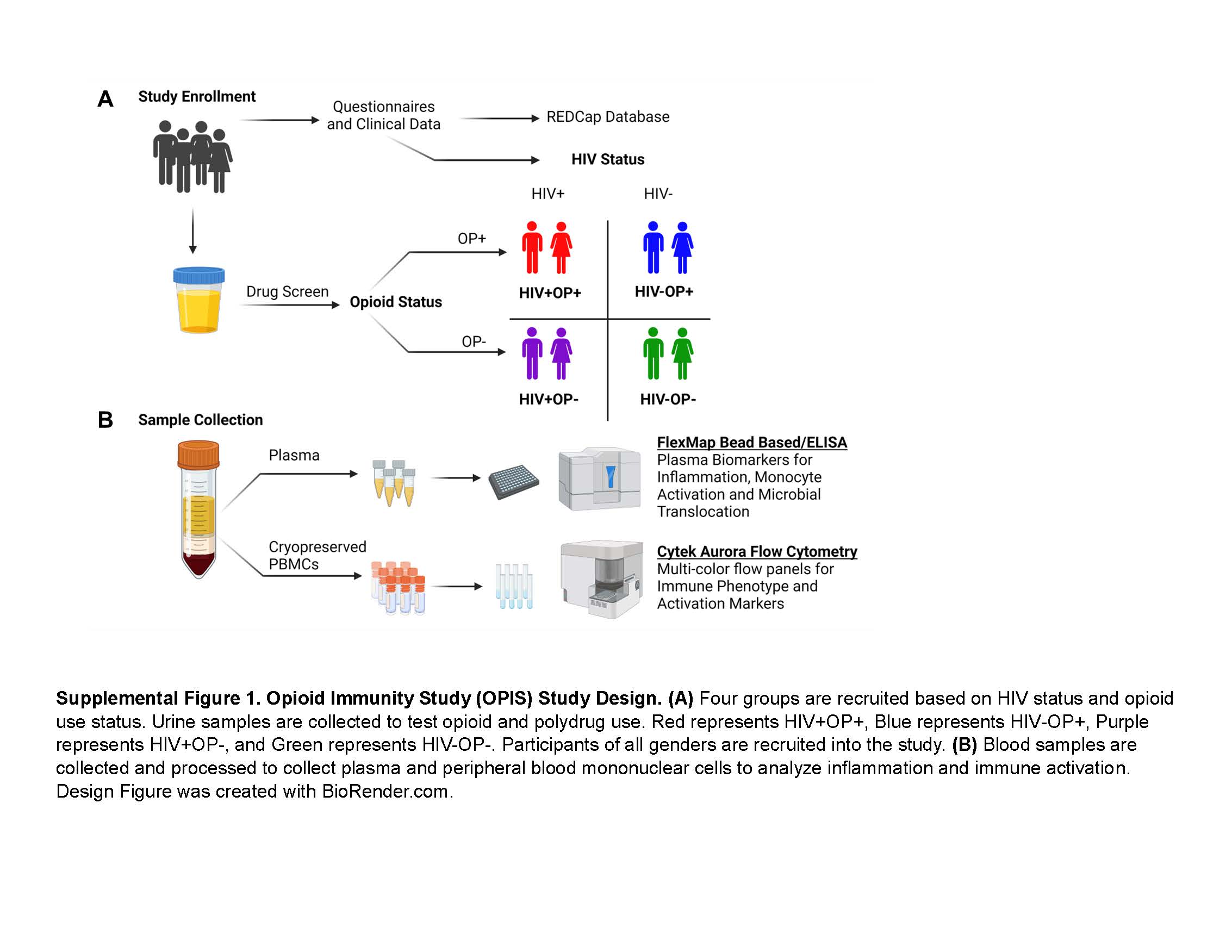

Supplement: Supplementary Figure 1 — Opioid Immunity Study (OPIS) Study Design. (A) Four groups are recruited based on HIV status and opioid use status. Urine samples are collected to test opioid and polydrug use. Red represents HIV+OP+, Blue represents HIV-OP+, Purple represents HIV+OP-, and Green represents HIV-OP-. Participants of all genders are recruited into the study. (B) Blood samples are collected and processed to collect plasma and peripheral blood mononuclear cells to analyze inflammation and immune activation. Design Figure was created with BioRender.com. [file Image_1.jpg]

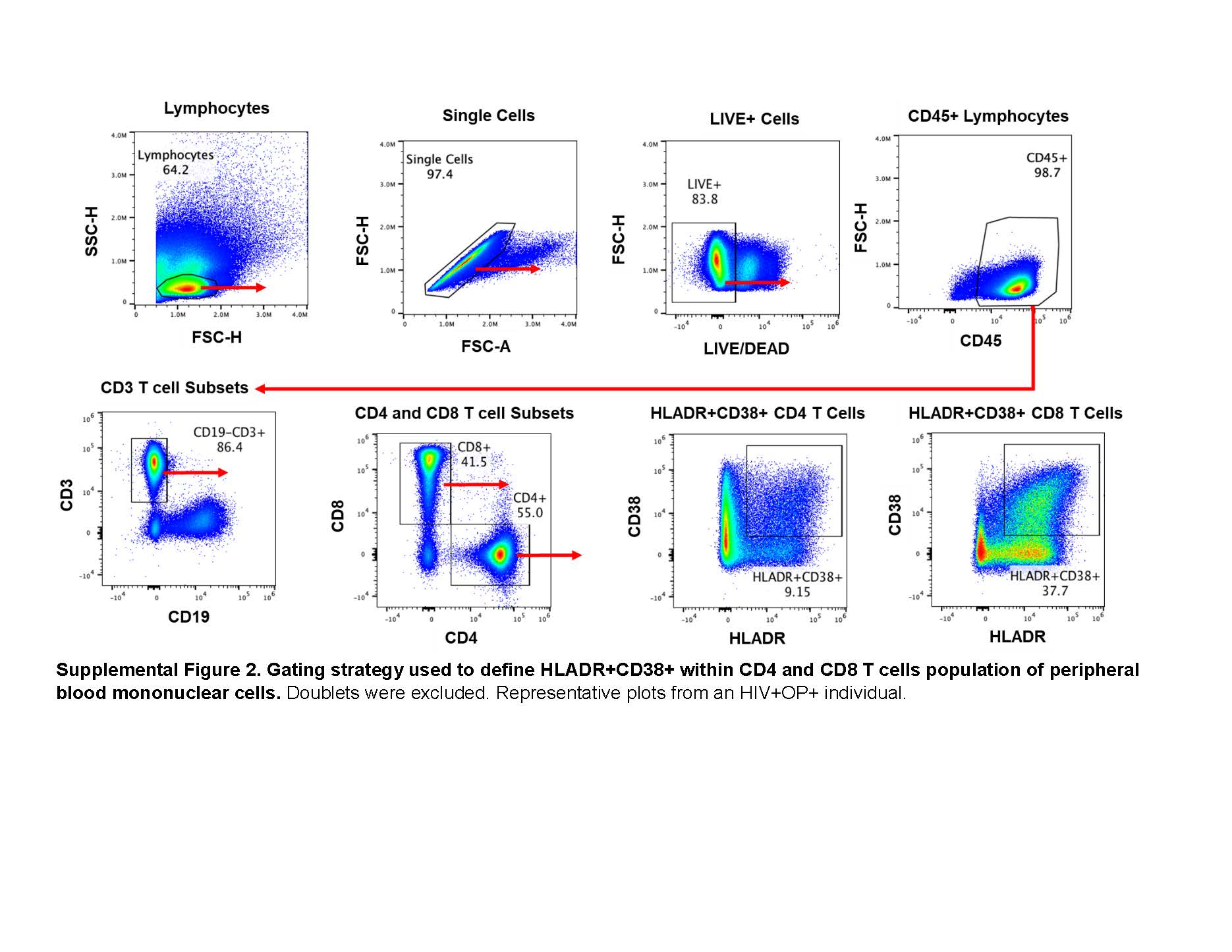

Supplement: Supplementary Figure 2 — Gating strategy used to define HLADR+CD38+ within CD4 and CD8 T cells population of peripheral blood mononuclear cells. Doublets were excluded. Representative plots from an HIV+OP+ individual. [file Image_2.jpg]

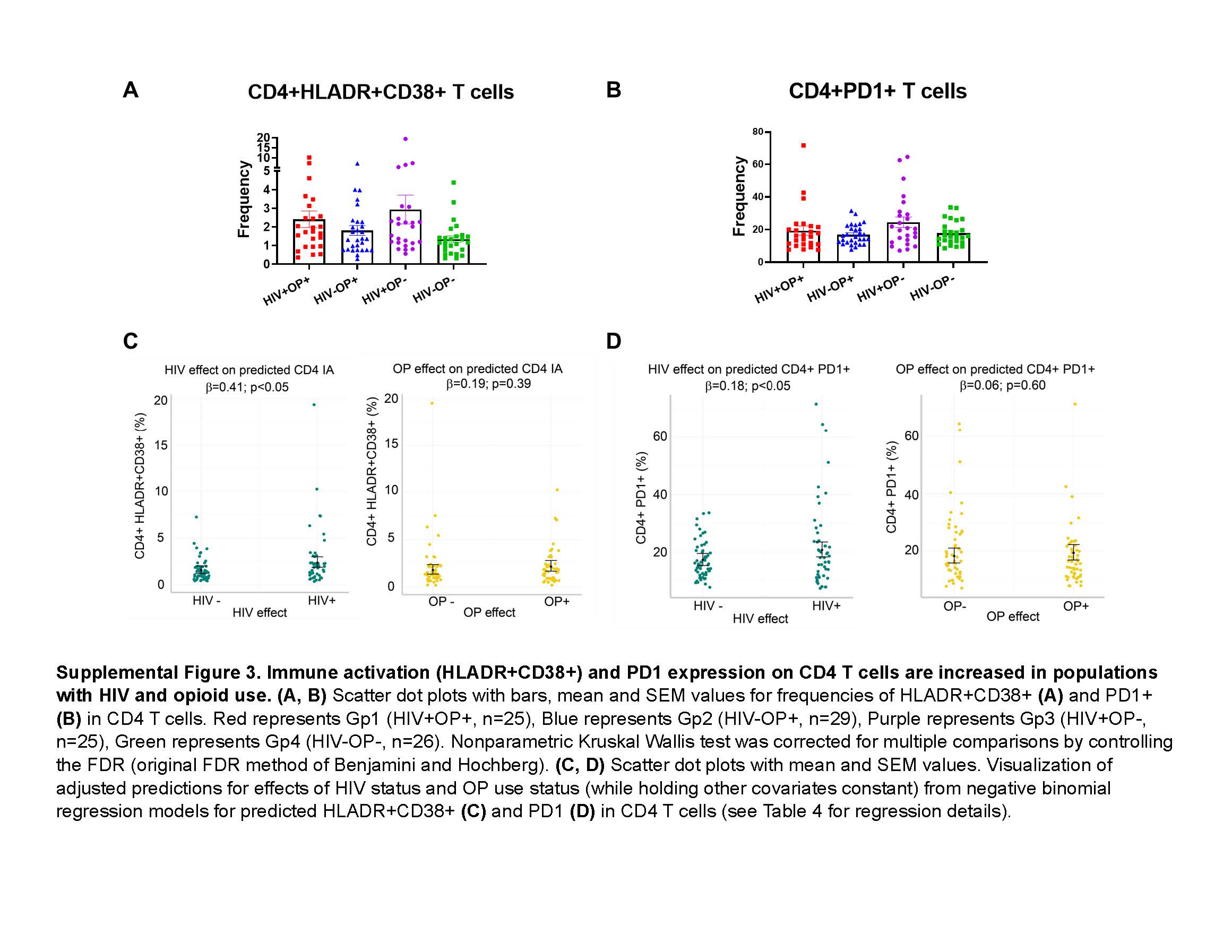

Supplement: Supplementary Figure 3 — Immune activation (HLADR+CD38+) and PD1 expression on CD4 T cells are increased in populations with HIV and opioid use. (A, B) Scatter dot plots with bars, mean and SEM values for frequencies of HLADR+CD38+ (A) and PD1+ (B) in CD4 T cells. Red represents Gp1 (HIV+OP+, n=25), Blue represents Gp2 (HIV-OP+, n=29), Purple represents Gp3 (HIV+OP-, n=25), Green represents Gp4 (HIV-OP-, n=26). Nonparametric Kruskal Wallis test was corrected for multiple comparisons by controlling the FDR (original FDR method of Benjamini and Hochberg). (C, D) Scatter dot plots with mean and SEM values. Visualization of adjusted predictions for effects of HIV status and OP use (while holding other covariates constant) from negative binomial regression models for predicted HLADR+CD38+ (C) and PD1 (D) in CD4 T cells (see Table 4 for regression details). [file Image_3.jpg]

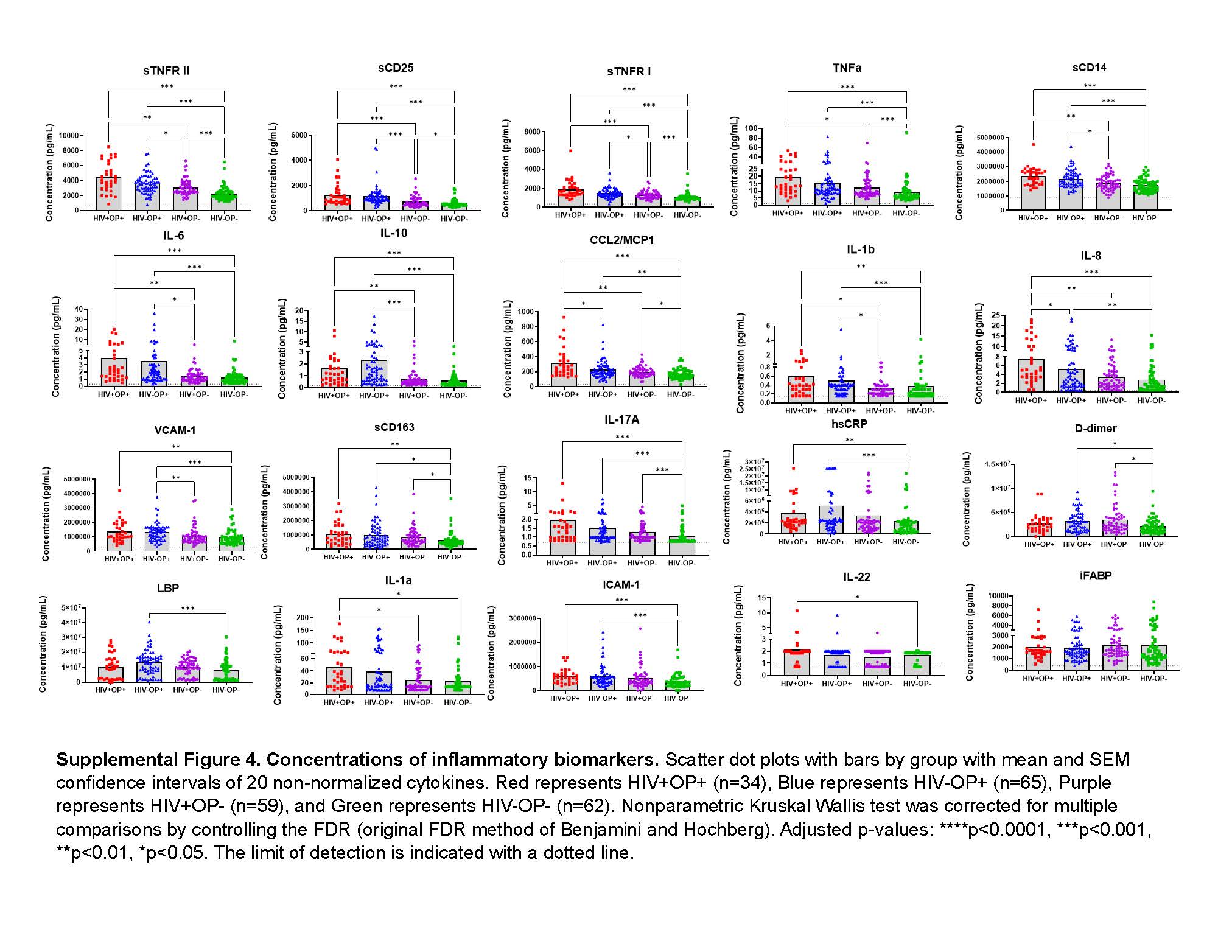

Supplement: Supplementary Figure 4 — Concentrations of inflammatory biomarkers. Scatter dot plots with bars by group with mean and SEM confidence intervals of 20 non-normalized cytokines. Red represents HIV+OP+ (n=34), Blue represents HIV-OP+ (n=65), Purple represents HIV+OP- (n=59), and Green represents HIV-OP- (n=62). Nonparametric Kruskal Wallis test was corrected for multiple comparisons by controlling the FDR (original FDR method of Benjamini and Hochberg). Adjusted p-values: ****p<0.0001, ***p<0.001, **p<0.01, *p<0.05. The limit of detection is indicated with a dotted line. [file Image_4.jpg]
