# Supplementary material for: Opioids exacerbate inflammation in people with well-controlled HIV
Source: Front Immunol. 2023 Nov 1;14:1277491. doi: 10.3389/fimmu.2023.1277491 (PMC10646416; doi:10.3389/fimmu.2023.1277491)
Supplement: Supplementary Table 1 — Panel of surface markers and reagents used in multiparameter flow cytometry. [file Table_1.pdf]

Surface Marker/Reagent	Color	Clone	Company	Catalog #
CD19	BUV395	SJ25C1	BD	563549
CD28	BV785	CD28.2	Biolegend	302950
CD3	BV570	UCHT1	Biolegend	300436
CD38	APC/Fire 810	HIT2	Biolegend	303550
CD4	BUV805	SK3	BD	612887
CD45	Alexa 532	HI30	Life Technologies	58-0459-42
CD57	BV421	NK-1	BD	563896
CD8	BB700	RPA-T8	BD	566451/2
CTLA-4	PE-Cy5	BNI3	BD	561717
HLA-DR	BUV661	G46-6	BD	612980
LAG-3	BV711	11C3C65	Biolegend	369320
PD-1	APC	EH12.2H7	Biolegend	329908
TIGIT	Alexa 647	A15153G	Biolegend	372724
TIM-3	APC-Cy7	F38-2E2	Biolegend	345026
Benzonase			Millipore	70664-3
FcX Blocker			Biolegend	422302
LIVE-DEAD Blue			Invitrogen	L23105
Paraformaldehyde Aqueous Solution			Electron Microscopy Sciences	15712-S
UltraComp Compensation Beads			ThermoFisher	01-2222-41

Supplemental Table 1. Panel of surface markers and reagents used in multiparameter flow cytometry.
