# Supplementary material for: Opioids exacerbate inflammation in people with well-controlled HIV
Source: Front Immunol. 2023 Nov 1;14:1277491. doi: 10.3389/fimmu.2023.1277491 (PMC10646416; doi:10.3389/fimmu.2023.1277491)
Supplement: Supplementary Table 2 — Multiple group comparisons of significant variables in cohort demographics and participant clinical profile, baseline urine drug screen (UDS). [file Table_2.pdf]

Significant Variables	Comparison among groups	HIV+OP+ vs HIV-OP+	HIV+OP+ vs HIV+OP-	HIV+OP+ vs HIV-OP-	HIV-OP+ vs HIV+OP-	HIV-OP+ vs HIV-OP-	HIV+OP- vs HIV-OP-
**Demographics**							
Age	$p < 0.001^a$	NS	$p < 0.01^a$	NS	$p < 0.001^a$	$p < 0.001^a$	NS
Gender	$p < 0.05^{b,\dagger}$	NS	NS	NS	$p < 0.01^c$	$p < 0.01^c$	NS
Race	$p < 0.0001^{b,\dagger}$	$p < 0.0001^c$	$p < 0.0001^c$	$p < 0.0001^c$	$p < 0.0001^c$	$p < 0.0001^c$	NS
HCV Ab seropositivity	$p < 0.0001^{b,\dagger}$	NS	$p < 0.0001^c$	$p < 0.0001^c$	$p < 0.0001^c$	$p < 0.0001^c$	NS
Baseline CD4 T-cell count (cells/uL)	$p < 0.001^a$	NS	NS	NS	$p < 0.05^a$	NS	$p < 0.001^a$
Baseline CD4/CD8 Ratio	$p < 0.001^a$	$p < 0.001^a$	NS	$p < 0.001^a$	$p < 0.001^a$	NS	$p < 0.001^a$
**Substance Use**							
# of Substances Used	$p < 0.001^a$	NS	$p < 0.001^a$	$p < 0.001^a$	$p < 0.001^a$	$p < 0.001^a$	NS
Injection Drug Use	$p < 0.0001^b$	$p < 0.05^c$	$p < 0.05^c$	$p < 0.01^c$	$p < 0.0001^c$	$p < 0.0001^c$	NS
Cocaine	$p < 0.0001^b$	NS	NS	$p < 0.01^c$	$p < 0.01^c$	$p < 0.0001^c$	NS
Benzodiazepine	$p < 0.01^b$	NS	$p < 0.01^c$	$p < 0.05^c$	NS	NS	NS
Ethyl-Glucuronide	$p < 0.05^b$	NS	NS	$p < 0.01^c$	$p < 0.05^c$	$p < 0.05^c$	NS

[a]Kruskal-Wallis; [b]Chi-Square; [c]Fisher’s Exact Test; NA=Not Applicable; NS=Not Significant

† = Participant removal due to small cell size (Gender (n=3), Race (n=13), HCV (n=1))

Supplemental Table 2. Multiple group comparisons of significant variables in cohort demographics and participant clinical profile, baseline urine drug screen (UDS)
